# Nursing Process in the Brazilian context: reflection on its concept and legislation

**DOI:** 10.1590/0034-7167-2021-0898

**Published:** 2022-05-11

**Authors:** Alba Lucia Bottura Leite de Barros, Amália de Fátima Lucena, Sheila Coelho Ramalho Vasconcelos Morais, Marcos Antônio Gomes Brandão, Miriam de Abreu Almeida, Marcia Regina Cubas, Tania Couto Machado Chianca, Viviane Martins da Silva, Maria Helena Baena de Moraes Lopes, Rosimere Ferreira Santana

**Affiliations:** I Universidade Federal de São Paulo. São Paulo, São Paulo, Brazil.; II Universidade Federal do Rio Grande do Sul. Porto Alegre, Rio Grande do Sul, Brazil.; III Universidade Federal de Pernambuco. Recife, Pernambuco, Brazil.; IV Universidade Federal do Rio de Janeiro. Rio de Janeiro, Rio de Janeiro, Brazil.; V Pontifícia Universidade Católica do Paraná. Curitiba, Paraná, Brazil.; VI Universidade Federal de Minas Gerais. Belo Horizonte, Minas Gerais, Brazil.; VII Universidade Federal do Ceará. Fortaleza, Ceará, Brazil.; VIII Universidade Estadual de Campinas. Campinas, São Paulo, Brazil.; IX Universidade Federal Fluminense. Rio de Janeiro, Rio de Janeiro, Brazil.

**Keywords:** Nursing Process, Legislation, Nursing, Education Nursing, Nursing Care, Standardized Nursing Terminology, Proceso de Enfermería, Legislación de Enfermería, Educación en Enfermería, Atención de Enfermería, Terminología Normalizada de Enfermería, Processo de Enfermagem, Legislação de Enfermagem, Ensino de Enfermagem, Assistência de Enfermagem, Terminologia Padronizada em Enfermagem

## Abstract

**Objectives:**

to reflect on the global understanding of the Nursing Process concept, with emphasis on the Brazilian context.

**Methods:**

a reflection article, aligned with the vision and expertise of researchers who are members of the Nursing Process Research Network.

**Results:**

the reflection is presented in two main topics: *The evolution of Systematization of Nursing Care X Nursing Process concepts and its consonance with national and international practices, and Brazilian legislation; The Nursing Process concept realignment in Brazilian legislation in line with current care, teaching and research practices*. **Final Considerations**: the reflections were oriented to the Nursing Process’ conceptual, normative and legal issues, including elements of its historical evolution, and, with that, pointed to the need to modify the Brazilian regulation on the Nursing Process.

## RESUMO

**Objetivos:**

refletir sobre a compreensão global do conceito de Processo de Enfermagem, com ênfase no contexto brasileiro.

**Métodos:**

ensaio reflexivo, alinhado à visão e expertise de pesquisadores membros da Rede de Pesquisa em Processo de Enfermagem.

**Resultados:**

a reflexão se apresenta em dois tópicos principais: *A evolução dos conceitos de Sistematização da Assistência de Enfermagem X Processo de Enfermagem e a sua consonância com as práticas nacional e internacional, e a legislação brasileira; Realinhamento do conceito Processo de Enfermagem na legislação brasileira em consonância com as práticas assistenciais, de ensino e pesquisa atuais*. **Considerações Finais**: as reflexões se orientaram às questões conceituais, normativas e legais do Processo de Enfermagem, incluindo elementos de sua evolução histórica, e, com isso, apontaram para a necessidade da modificação da regulamentação brasileira sobre o Processo de Enfermagem.

## RESUMEN

**Objetivos:**

reflexionar sobre la comprensión global del concepto de Proceso de Enfermería, con énfasis en el contexto brasileño.

**Métodos:**

ensayo reflexivo, alineado con la visión y experiencia de investigadores integrantes de la Red de Investigación del Proceso de Enfermería.

**Resultados:**

la reflexión se presenta en dos temas principales: *La evolución de los conceptos de Sistematización de la Atención de Enfermería X Proceso de Enfermería y su consonancia con las prácticas nacionales e internacionales, y la legislación brasileña; La realineación del concepto de Proceso de Enfermería en la legislación brasileña de acuerdo con las prácticas actuales de atención, enseñanza e investigación*. **Consideraciones Finales:** las reflexiones se orientaron a las cuestiones conceptuales, normativas y legales del Proceso de Enfermería, incluyendo elementos de su evolución histórica, y, con eso, apuntaron para la necesidad de cambiar la regulación brasileña sobre el Proceso de Enfermería.

## INTRODUCTION

The Nursing Process (NP) term was introduced in the 1950s in the United States of America, referring to a process that, through the scientific method, combines the most desirable elements of the art of nursing with the most relevant elements of theoretical systems. It encompassed an interpersonal approach/approach and the problem-solving method for decision-making in nursing. Gradually, with its application in practice, its steps were thickened and refined, in order to support individualized care with greater quality and safety. This movement was accompanied by Brazilian nursing, about two decades later, by the theoretical and practical reflections of Wanda de Aguiar Horta, published throughout the 1970s^(1)^.

To implement the NP in our country, Horta carried out missions in different cities and health units, but encountered difficulties, such as nursing staff sizing, limited availability of material and hierarchy of the task-centered Fordist managerial model of nursing. As there is a culture of nurses’ work in the management of nursing actions, the change to a model of clinical work centered on the person and on professional results required the rearrangement of nursing care systems.

It was only at the end of the 1980s that NP-related activities began to be supported in Brazil by professional nursing legislation in the regulation of prescription and nursing consultations as exclusive attributions of nurses^(2)^. In the early 2000s, the Federal Nursing Council (COFEN - *Conselho Federal de Enfermagem*) published Resolution 272/2002, introducing the term Systematization of Nursing Care (SNC), in addition to NP^(3)^. This Resolution indicated that the SNC should be formally recorded in patients’/clients’/users’ medical records, consisting of nursing history, physical examination, nursing diagnosis, nursing care prescription, nursing care evolution and nursing report, even if without the construction of adequate conceptual limits.

In an attempt to minimize the mistake made, COFEN Resolution 272/2002 was revoked and replaced by 358/2009, which sought to clarify the difference between SNC and NP^(4)^. Although it is possible to perceive an evolution in the sense of clarifying, arranging and offering consistency to the different concepts, the ambiguity in its interpretation remained present in the written and oral communication of many nurses.

Such findings have generated debate among academic and service nurses. More recently, they have been shared by researchers from the Nursing Process Research Network (RePPE - *Rede de Pesquisa em Processo de Enfermagem*), generating discussions of a semantic-conceptual and operational nature concerning NP. RePPE is made up of researchers from higher education and health institutions in different states of Brazil and international collaborating centers that have studied the NP theme in order to generate, synthesize and share knowledge on the subject, its theoretical frameworks and classification systems.

Reflections on NP’s conceptual nature, resulting from activities carried out by RePPE members, include: development of documents on elements of nursing practice (diagnoses, results and interventions), based on standardized language systems (SLP), to guide nursing practice in care settings for patients with COVID-19, published in a scientific journal^(5)^; studying the subject in a network, supported by its researchers, offered in Graduate Nursing Programs throughout Brazil; strengthening partnerships in research projects; advising on education and health services; and presentations at national and international events on NP.

From all these RePPE activities, concerns emerged centered on the following question: after almost 20 years of the enactment of COFEN Resolution 272/2002^(3)^ and more than a decade of its revocation by COFEN Resolution 358/2009^(4)^, what reflections can be elaborated on the global understanding of NP in Brazil?

## OBJECTIVES

To reflect on the global understanding of the NP concept, with emphasis on the Brazilian context.

## METHODS

This is a reflection article, aligned with the vision and expertise of RePPE member researchers. The main points of discussion were derived from the reflective analysis supported by the national and international literature and the Brazilian professional nursing legislation. Discussions are organized into topics: *The evolution of Systematization of Nursing Care X Nursing Process concepts and its consonance with national and international practices, and Brazilian legislation; The Nursing Process concept realignment in Brazilian legislation in line with current care, teaching and research practices*.

## DISCUSSIONS ARISING FROM THE REFLECTION

### The evolution of Systematization of Nursing Care X Nursing Process concepts and its consonance with national and international practices, and Brazilian legislation

When reflecting on aspects that influenced the connotative-interpretative meaning of synonymy or distinction between SNC and NP terms, the need to revisit historical aspects and analyze elements that contribute to the construction or strengthening of nursing professional identity emerges^(6)^. In view of this dynamic evolutionary process, it is understood that it is relevant to dialogue about SNC and NP traditions and translations with history and projections of NP generations.

NP generations were described chronologically, the first between the period 1950 and 1970, the second between 1970 and 1990, and the third between 2000 and 2010, with estimated directions for possible future routes of the fourth, fifth and sixth generations in the period between 2020 and 2060^(7)^.

The first NP generation was oriented to look for nursing problems and to the intervention steps and the justification for its accomplishment. The second generation, marked by NP structuring, changes the process from the four initial steps to five, including nursing diagnosis. This new stage gave rise to the idea of building SLP, in order to describe the elements of practice, such as nursing diagnoses, pointing to dismemberments that favored clinical reasoning and the search for effective results, which characterizes the next NP generations^(7)^.

From this conceptualization process of NP evolution in generations, one can imagine the challenge to training nurses and arranging health services to advance, in addition to the possible difficulty of dialogue between professionals trained in the first generation with those of later generations, coexisting in the same care setting.

In Brazil, the knowledge generated in the first two NP generations formed the basis for the first discussions on the subject. In 1971, Horta defined NP as the “dynamics of systematized and interrelated actions, aiming at assistance to the individual, family and community”, organizing it into six stages - nursing history, nursing diagnosis, nursing therapeutic plan - later called care, nursing care plan, nursing evolution and nursing prognosis^(1)^. From the NP, other concepts were introduced: nursing care, defined as NP application by a nurse, and nursing care, corresponding to nurses’ planned, deliberate or autonomous action, resulting from the perception, observation and analysis of human beings’ behavior, situation or condition. There is also the operational concept of nursing consultation, which consists of applying the NP to the supposedly healthy individuals undergoing outpatient follow-up^(1-3)^.

The advance of nursing professionalization in Brazil, added to precursor studies on NP, to the efforts between the Union, the Brazilian Nursing Association (ABEn - *Associação Brazileira de Enfermagem*) and the COFEN/Regional Nursing Council (COREN - *Conselho Regional de Enfermagem*) system, led to the approval of Law 7,498/1986, which regulated, among activities, the prescription and nursing consultation as exclusive duties of nurses^(2)^.

At the same time, the creation and expansion of graduate nursing programs were observed in the country. Teaching NP and its stages influenced a generation of nurses aware of the importance of using this theoretical-methodological framework. From this, articles, books, monographs, dissertations and theses produced arose, which also began to impact undergraduate teaching and practice of Brazilian students.

Although, until the beginning of the 2000s, NP evolution in Brazil had more slowly followed the characteristics described at the international level, in this period there was a search for advancement of legal aspect, culminating in COFEN Resolution 272/2002, which had on SNC in health institutions, defining it as a private activity of nurses^(3)^. In this context, understanding the NP and its stages had not advanced, and the SNC socialization had an impact on nursing practice, revealing a conflict in the daily vocabulary for using the two terms.

COFEN Resolution 272/2002, having SNC as a private activity of nurses, indicated that it would use scientific work method and strategy to identify health/disease situations, subsidizing nursing care actions that could contribute to the promotion, prevention, recovery and rehabilitation of individuals’, family’s and community’s health^3^. Thus, nurses were privately responsible for NP implementation, planning, arrangement, execution and assessment. The SNC should be formally recorded in patients’/clients’/users’ medical record, consisting of a nursing history, physical examination, nursing diagnosis, nursing care prescription, evolution of care, in addition to nursing report. SNC, NP and nursing care concepts are used without a clear distinction or indication of the relationship between them^(3)^. Thus, the consequence would be to ask: are these terms synonymous or are they distinct constructs?

Literature review^(8)^ aimed to contribute to the answer to the question presented, explaining different currents of national thought on the subject. Thus, it described that the current that addressed the terms as distinct defined SNC as “nursing work arrangement, in terms of method, personnel and instruments, in order to make it possible to carry out the NP”. At the same time, this current defined NP as a “methodological and systematic instrument for providing care, which served the intellectual activity of nurses and provided a guide for a particular style of judgment”. In other words, NP would be one of the methods for applying SNC and would then be integrated into its concept, making the conceptual distinction between them even more difficult.

Then, additional questions were posed to the reflection and interpretation of COFEN Resolution 272/2002, which could lead to the understanding that SNC and NP concepts were being treated as synonyms, and, if they were not, what would fact would constitute each of them? In view of this, a movement of scholars in the country culminated, in 2009, with the formation of a working group with members of ABEn and COFEN, to review COFEN Resolution 272/2002^(3)^, then create COFEN Resolution 358/2009^(4)^.

In COFEN Resolution 358/2009, SNC was described as the one that “arranges professional work in terms of method, personnel and instruments, making it possible for NP to be operational”^(4)^. However, despite starting with SNC’s conceptual description, this resolution deliberates, in its entirety, on NP aspects and stages and how it should be performed in all environments of professional nursing care, including the requirement for theoretical support. Note that, except for this definition of SNC, comprehensive in its purpose, nothing is informed about its nature in terms of attributes. It is not described about its constitution or form of implementation, which would communicate the operational definition that the autarchy (COFEN) expects from professionals in relation to its compliance, i.e., it does not present in detail what “people, instruments and method”^(4)^.

In turn, NP is detailed and operationally defined in each of its five stages, which must be interrelated, interdependent and recurrent, namely: nursing data collection (or nursing history); nursing diagnosis; nursing planning; nursing implementation and assessment^(4)^.

It should be noted that, at the time of publication of COFEN Resolution 358/2009^(4)^, the difficulties in implementing NP were already related to the institutional and professional work process arrangement, in addition to the specificities inherent to the method. Such difficulties make it possible to observe NP use in an incomplete or inadequate way, not identifying the implementation of all its stages, in the vast majority of services.

The real limitation of English language appropriation by nurses is added to the problem of NP application, which makes it difficult, until the present day, the globalization of Brazilian nursing knowledge and its internationalization. However, the training of master’s and PhD holders with expertise in the subject, promoted by the graduate course in nursing in Brazil, has broadened and deepened knowledge in the area, although there are ongoing challenges to conceptualize and reconceptualize professional practice. Although there was, and still is, resistance from some nurses and professors, others persisted in teaching and researching the topic. Model units were established, mainly in university hospitals, seeking to highlight the impact of NP use on the quality of teaching and care.

Thus, despite the attempt at conceptual adequacy of SNC and NP in COFEN Resolution 358/2009^(4)^, the way in which the terms were presented continues to contribute to the persistence of the difficulty of Brazilian nursing professionals in understanding and differentiating them. It is noteworthy that the term SNC is used only in Brazil and, many times, its use has been used as a synonym for NP^(6)^. As a result, this conceptual use can attribute to Brazilian nursing an anachronism in its knowledge in a globalized world, in addition to making it difficult to understand and consume the knowledge produced.

Since the 2010s, what can be characterized as a fourth NP generation^(7)^ in the search for relevant scientific evidence from studies of validity and reliability of diagnoses and results or the effectiveness of nursing interventions^(9)^. These results generate nursing databases that, together with other graduate nursing productions, strengthen evidence-based practice. Furthermore, there is a trend in current Brazilian publications for the exclusive NP concept use, including critical thinking and use of SLP in the documentation of its different stages^(10)^, which converges with the latest NP generations^(7)^. In this, Brazilian nursing has stood out, being one of the most published on the subject in the world.

### The Nursing Process concept realignment in Brazilian legislation in line with current care, teaching and research practices

In our view, SNC and NP concepts are distinct from a theoretical-conceptual and operational point of view. Based on the discussions presented in the previous topic, the researchers believe that it is appropriate to think that COFEN Resolution 358/2009^(4)^ needs to be revised, in order to update it in line with current knowledge.

It seems that the most appropriate would be to use only the concept of NP, as is the case worldwide. Alternatively, it would be necessary to expand the SNC concept, detailing its definition, forms of application and presentation of the explicit differentiation of the NP. To this end, constitutive and operational definitions that can explain concepts’ attributes are essential, in order to avoid overlaps or indistinctions.

Thus, it is understood that theoretical, scientific and legal support are necessary so that SNC’s conceptual and operational dimensions become clear and consensual for the professional category. It is noteworthy that, in this case, the normative descriptions of SNC definition and operationalization must be sufficiently detailed, to allow measuring satisfaction, professional autonomy, visibility, credibility, patient and professional safety, in addition to the result of nursing work.

There are several factors that affect NP implementation, as well as different methods and characteristics of education for the NP in the world, which is not, therefore, something peculiar to our country. However, there is no proposition of other similar concepts to organize and/or elaborate a practice documentation in the specificity of the NP. Thus, it is necessary to reflect on the routes and directions in the Brazilian context and reinforcing the need for a regulation that helps the conceptual delimitation and operational necessary for Brazilian nursing practice.

As a final reflection of this manuscript, it is understood the need to discuss the team professionals’ attributions in the documents. Notably, nurses must privately, among others, direct the nursing body, guide and supervise work, and nursing programming, which includes prescribing care. The exclusive duties of nurses do not exclude nursing technicians, including participation in the NP. Thus, based on current legislation, the question is in which NP stages would nursing technicians’ attributions be covered?

In this regard, COFEN Resolution 58/2009, in one of its articles, explains that the participation of nursing technicians in NP execution is their responsibility, under nurses’ supervision and guidance^(4)^. However, it is assumed that such attributions should be more detailed in a review of the Resolution, in order to avoid misinterpretations.

These considerations, punctuated in a reflective way, are intended to return to the debate on professional identity. We understand that NP allows us to distinguish ourselves from other professionals, and, when analyzed, from the perspective of a unifying matrix of the professional group, it encompasses objectives, ethical and legal frameworks and the set of actors that share a common reference, in order to build and rebuild our knowledge and practices. One can understand that the primary identity of nursing is the care implemented through the NP, which guides doing and thinking, enabling professional practice documentation^(6)^.

## FINAL CONSIDERATIONS

The reflections presented were oriented to NP’s conceptual, normative and legal issues, including elements of its historical evolution, and, with that, pointed to the need to modify the Brazilian regulation on it. It is hoped that the discussions brought about from the reflection can collaborate to obtain conceptual clarity and to advance in the recognition of NP’s properties and stages, which involve clinical reasoning and decision-making involved in its use.

The authors concluded, through reflection, that it would be advisable to carry out a review and modification of the Brazilian regulation on the subject, in order to produce alignment with the current understanding of researchers about NP.

It is assumed that the clarification of the NP characteristics and limits and the consequent clarification in the legislation will facilitate disseminating results of Brazilian studies on the subject in congresses or in articles published in international journals. For the service, concept-legislation alignment can bring operational benefits in NP introduction and implementation in health units.

Finally, it is argued that the NP should be taught based on current legislation in the country and on updated knowledge, transversally, in undergraduate courses, and in technical nursing courses, longitudinally, in professional life, constituting a standard of nursing practice.
